# Therapeutic Potential of *Hericium erinaceus* for Depressive Disorder

**DOI:** 10.3390/ijms21010163

**Published:** 2019-12-25

**Authors:** Pit Shan Chong, Man-Lung Fung, Kah Hui Wong, Lee Wei Lim

**Affiliations:** 1 School of Biomedical Sciences, Li Ka Shing Faculty of Medicine, The University of Hong Kong, Hong Kong, China; u3005073@connect.hku.hk (P.S.C.); fungml@hku.hk (M.-L.F.); 2 Department of Anatomy, Faculty of Medicine, University of Malaya, Kuala Lumpur 50603, Malaysia

**Keywords:** *Hericium erinaceus*, Lion’s mane mushroom, depression, antidepressant, mood disorders

## Abstract

Depression is a common and severe neuropsychiatric disorder that is one of the leading causes of global disease burden. Although various anti-depressants are currently available, their efficacies are barely adequate and many have side effects. *Hericium erinaceus,* also known as Lion’s mane mushroom, has been shown to have various health benefits, including antioxidative, antidiabetic, anticancer, anti-inflammatory, antimicrobial, antihyperglycemic, and hypolipidemic effects. It has been used to treat cognitive impairment, Parkinson’s disease, and Alzheimer’s disease. Bioactive compounds extracted from the mycelia and fruiting bodies of *H. erinaceus* have been found to promote the expression of neurotrophic factors that are associated with cell proliferation such as nerve growth factors. Although antidepressant effects of *H. erinaceus* have not been validated and compared to the conventional antidepressants, based on the neurotrophic and neurogenic pathophysiology of depression, *H. erinaceus* may be a potential alternative medicine for the treatment of depression. This article critically reviews the current literature on the potential benefits of *H. erinaceus* as a treatment for depressive disorder as well as its mechanisms underlying the antidepressant-like activities.

## 1. Introduction

Major depressive disorder, also known as depression, is a common neuropsychiatric disorder that affects more than 300 million people of all ages [1] and is one of the leading causes of global disease burden [2]. The common signs and symptoms of depression include loss of interest in daily activities, difficulty concentrating and making decisions, fatigue, sleep problems, overeating or appetite loss, pessimism, hopelessness, persistent sadness, and restlessness [3,4]. Untreated depression could lead to suicidal thoughts or suicide attempts [3,5]. Suicide is the second leading cause of death in young adults worldwide [1], and approximately 800,000 cases of suicide are reported annually [1].

In the 1950s, pharmacotherapy has become the main treatment for depression since the introduction of the first generation of antidepressants, which are monoamine oxidase inhibitors (iproniazid) and tricyclic antidepressants (imipramine) that have been on the market the longest [6,7]. In the 1980s, the second generation of antidepressants, including selective serotonin reuptake inhibitors (e.g., fluoxetine, sertraline, and paroxetine) and selective serotonin noradrenaline reuptake inhibitors (e.g., venlafaxine, duloxetine, and desvenlafaxine), were introduced as a safer class of antidepressants [6]. Other antidepressants that are prescribed less often include serotonin 5-HT2C receptor antagonists (e.g., olanzapine), alpha-2 blockers (e.g., atipamezole), melatonin receptor agonists (e.g., ramelteon), and selective noradrenaline/dopamine reuptake inhibitors (e.g., nomifensine). Although many types of antidepressant drugs are available, their overall efficacy is still barely satisfactory [7,8]. Studies of antidepressants in adults with moderate or severe depressive disorder revealed that currently available antidepressants could only relieve symptoms of depression in about 20% of patients [8]. Furthermore, antidepressant medication often needs to be administered continuously for years to prevent relapse [9]. Besides, more than 50% of antidepressant users reported that they experienced side effects, including headaches, dry mouth, anxiety, dizziness, weight gain, decreased interest in sex, as well as a loss of ability to have an orgasm or an erection [10,11]. The side effects can often lead to failure in the administration of antidepressants in depressive patients [12,13].

**Figure 1:**
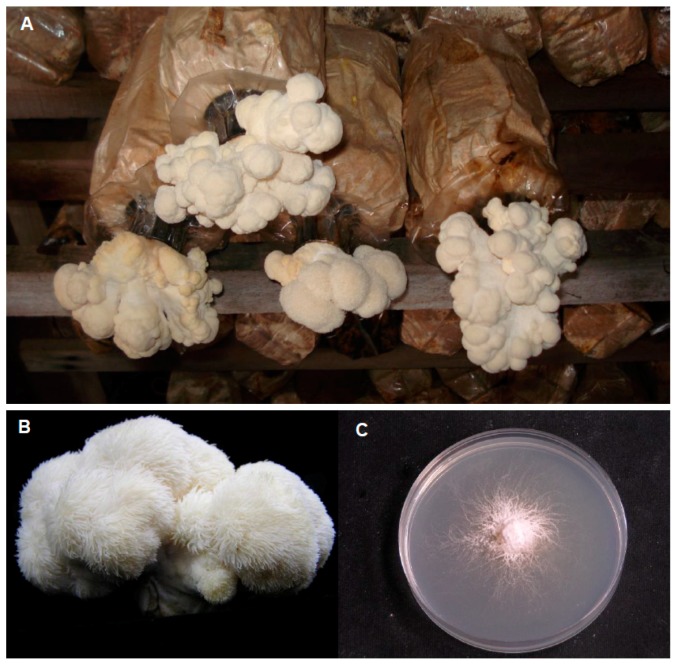
Fruiting bodies cultivated at the tropical climate in Malaysia (**A**,**B**) and mycelia (**C**) of *Hericium erinaceus* grown on potato dextrose agar.

Herbal medicine can be a cost-effective complementary and alternative medicine for the treatment of depressive disorders, generally with fewer side effects and limited comparative efficacy to conventional antidepressants, as well as it is well-tolerated by depressive patients [14,15]. Mushrooms are functional foods with high nutritional values and are great sources for novel therapeutic compounds [13,16]. *Hericium erinaceus* is a medicinal-culinary mushroom widely found in East Asian countries and is commonly known as lion’s mane mushroom, Yamabushitake, or monkey’s head mushroom [17]. *Hericium erinaceus* has a long history as a medicine [17] and has been found to promote positive nerve and brain health. It has great potential in treating neurological disorders as it contains neurotrophic compounds that can pass through the blood–brain barrier [18,19]. Bioactive compounds extracted from its fruiting body or mycelium (Figure 1) have been demonstrated to possess antioxidative [20], antidiabetic [21], anticancer [22,23], anti-inflammatory [24], antimicrobial [23], antihyperglycemic [25], and hypolipidemic properties [26]. Moreover, *H. erinaceus* has been used to treat cognitive impairments [27], Alzheimer’s disease [28], Parkinson’s disease [29], ischemic stroke [30], and presbycusis [14]. Recently, the present research on *H. erinaceus* has been focused on its antidepressant-like effects for the treatment of depressive disorder [31,32,33]. Up to date, no review concerning the antidepressant effects of *H. erinaceus* is available. The aim of this review is to critically review the current literature on the potential antidepressant effects of *H. erinaceus* as a treatment for depressive disorder as well as its possible mechanisms underlying the antidepressant-like responses.

## 2. Pathophysiology of Depression

Depression is a complex disorder and its etiology is believed to be heterogeneous with many causes and contributing factors. The pathophysiology of depression is unclear, but it is believed to involve neurodegeneration and neurobiological changes [34]. The following section discusses the hypotheses of the pathophysiology of depression relating to the therapeutic potential of *H. erinaceus*.

### 2.1. Monoamine Hypothesis

**Figure 2:**
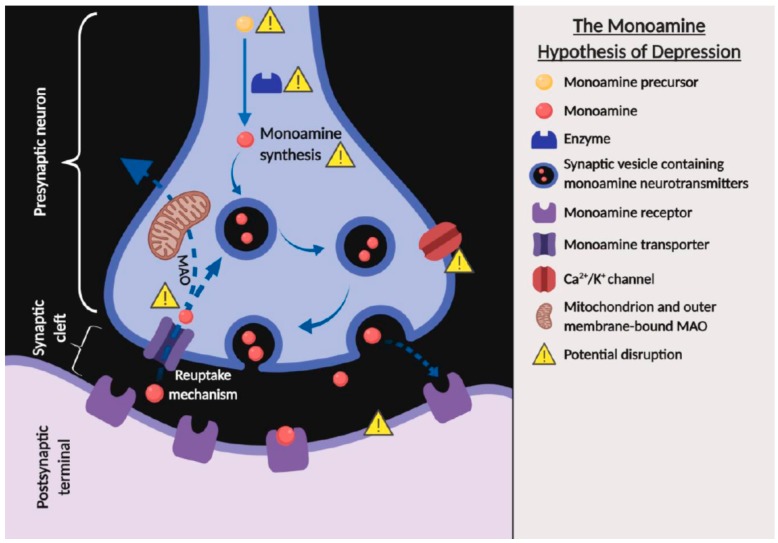
The monoamine hypothesis of depression showing the potential factors that can cause a deficiency in the transmission within the monoamine systems (created with BioRender.com). The solid arrows indicate the flow of synaptic vesicles containing monoamine neurotransmitters. The dotted arrows indicate the release or reuptake of the monoamine neurotransmitters across the terminal of presynaptic neuron.

The monoamine hypothesis of depression suggests that the major signs and symptoms of depression are associated with a deficiency in the transmission within the monoamine systems, including norepinephrine, serotonin, or/and dopamine [35,36]. The deficiency in the transmission of monoamine neurotransmitters can be caused by several factors, including the deficiency or malfunctioning in monoamine precursors, enzymes, receptors, transporters; monoamine synthesis; high level of monoamine oxidase function; and reduction in exocytosis that are indirectly modulated by the chemically-gated channels (Figure 2) and clinical in vivo findings have provided much evidence to support the monoamine hypothesis [35,36,37]. Reserpine, a drug that was commonly used to treat schizophrenia and hypertension in the early 1950s, and clinical observations have revealed that the administration of reserpine depleted presynaptic stores of norepinephrine, serotonin, and dopamine, which led to a syndrome resembling depression in some patients [37,38,39]. Interestingly, in vivo studies found that animals treated with reserpine exhibited depressive-like behavior, which is in line with clinical findings [40,41]. In contrast, iproniazid, a drug formulated in the 1950s to treat tuberculosis was reported to inhibit the metabolic enzyme monoamine oxidase (MAO), leading to increased extracellular levels of norepinephrine and serotonin in the brain and ultimately resulting in a euphoric and hyperactive state in a subset of patients [42]. Iproniazid and other monoamine oxidase inhibitors were subsequently found to be effective in improving symptoms of depression, which strongly supports the monoamine hypothesis of depression. Similarly, antidepressant-like effects were also observed with the oral administration of *H. erinaceus* in depressive-like animals, and it was found to restore expression levels of norepinephrine, serotonin, and dopamine [31].

### 2.2. Neurotrophic/Neurogenic Hypothesis

The neurotrophic hypothesis of depression involves the neuroplasticity and adaptation of the nervous system, and the inability of the nervous system to respond or adapt appropriately to aversive stimuli or stress resulting in depression. Anti-depressant drugs that stimulated appropriate adaptive responses were able to alleviate the symptoms of depression [42]. This hypothesis is associated with the neurogenic hypothesis that is based on the concept that neurogenesis is negatively regulated during a stressful condition, which can be positively regulated by antidepressant treatments. Pre-clinical and clinical findings showed that depression and stress were associated with volumetric decreases in the hippocampus of adult patients, whereas chronic anti-depressant treatment was able to increase the proliferation and survival rate of hippocampal neural progenitors [43,44,45,46]. The hippocampus is a neurogenic area in the brain that plays a critical role in learning, memory, and emotion. Neurotrophic factors are growth factors in the nervous system that play a role in modulating the plasticity of neuronal cells [47,48]. Brain-derived neurotrophic factor (BDNF) is one of the neurotrophic factors that are highly associated with suicidal and depressive behaviors [49,50,51]. Although animal studies showed a decrease in BDNF level was not sufficient to produce depressive-like behaviors, clinical evidence showed that there was a reduction in BDNF levels with neuronal dysfunction in the brains of patients with major depressive disorder [52,53,54]. Anti-depressant treatments that restored or increased BDNF levels were able to alleviate symptoms of depression [51,53,55]. BDNF plays a role in activity-dependent neuroplasticity including cognitive function and memory. Increased BDNF expression level is known to induce several types of neuroplasticity, including synaptogenesis, adult-neurogenesis, and neuronal maturation [56]. BDNF functions by binding to its receptors, including tropomyosin receptor kinase B (TrkB) and pan 75 neurotrophin receptor (p75^NTR^), a low-affinity receptor. The binding affinity of p75^NTR^ towards BDNF was found to be increased with less Trk receptors or Trk inactivity [57]. This interaction can induce neuronal apoptosis in oligodendrocytes, vascular smooth muscle cells, and neuronal cells [47]. The alteration in BDNF-TrkB signaling is believed to be involved in the pathogenesis of depression, which could be targeted therapeutically.

### 2.3. Inflammatory Hypothesis

Depressive disorder was found to link with an increase in expression of various central and peripheral proinflammatory cytokines, including tumor necrosis factor α (TNF-α) and interleukin-1, interleukin-6 (IL-1, IL-6), and interferon- α and γ [58,59,60,61]. Evidence from an in vivo study shows that the concentration of IL-1 and IL-2 was increased in the rat model of depression subjected to chronic mild stress [62]. Furthermore, animal models of inflammation-associated depression are often generated through the administration of cytokines or cytokine inducers, including IL-6 and lipopolysaccharide (LPS) [63,64,65]. The administration of IL or LPS, and further exposure to inflammatory induction could develop depressive-like syndrome and behavior including anorexia, anhedonia, and reduction in locomotor activity [66,67,68]. An in vivo study also showed that stress stimulus significantly increases the level of proinflammatory cytokines including TNF-α and IL-18 in the prefrontal cortex and hippocampus [69]. The inflammatory hypothesis is further supported by a clinical trial whereby an acute increase of depressive-like symptoms was reported in healthy volunteers administered with LPS [66]. Patients with the first episode of depression and those who experiencing recurrent depressive disorders were reported to have no difference in the concentration of IL-1, IL-6, and IL-10, suggesting that altered expression of the proinflammatory cytokine is a constant characteristic for depression [70]. Additionally, TNF-α involves in the onset of the glucocorticoid resistance and activation of the hypothalamo–pituitary–adrenocortical axis, induces excessive reuptake of monoamines and stimulates the indoleamine 2,3-dioxygenase which result in tryptophan and serotonin depletions [71,72]. Thus, the inflammatory pathway could be a potential target for the treatment of depression.

## 3. *Hericium erinaceus* Ameliorates Depressive-Like Behaviors

### 3.1. Pre-Clinical Studies

The therapeutic effects of *H. erinaceus* have been widely studied in several neurological diseases. However, not many studies have investigated its use in mental disorders. This review summarizes the behavioral and physiological effects of different *H. erinaceus* extracts in the studies of depression (Table 1).

Amycenone is an *H. erinaceus* extract that obtained from the fruiting body through a patented process, which contains 0.5% hericenone and 6% amyloban [73]. In 2015, Yao et al. reported the antidepressant-like and anti-inflammatory effects of amycenone in an animal model of depression with LPS-induced inflammation [33]. Amycenone was administered orally to mice 60 min before the intraperitoneal injection of LPS, and behavioral tests were performed 24 h after LPS injection. They found that acute treatment of 200 mg/kg amycenone significantly reduced the depressive-like behaviors with significant reduction of the LPS-induced immobility in both the forced swim and tail suspension tests. These results demonstrated the antidepressant-like effects of amycenone in an animal model of LPS-induced inflammation depression, suggesting its neuroprotective effects against inflammation-associated depression. However, treatment of depression usually required long-term administration of antidepressant and acute treatment might not provide a long-term therapeutic effect, which might eventually trigger a recurrence. The experiments conducted by Yao et al. could be improved by prolonging the study to examine the long-term antidepressant effects of amycenone.

The study by Ryu et al. in 2018 investigated the antidepressant and anxiolytic effects of *H. erinaceus* ethanolic extract in adult mice [32]. They found that chronic administration of a high dose (60 mg/kg) *H. erinaceus* extract significantly reduced the time spent in the peripheral region of the open field test, suggesting a potential anxiolytic effect. Furthermore, immobility time was significantly reduced in both the tail suspension test and forced swim test, indicating an anti-depressant-like effect. However, the animal model in this study used naive animals that were not pre-exposed to stress. The effectiveness of *H. erinaceus* as an antidepressant could vary between naive and depressed subjects, as they have different behaviors and physiological responses. These results may be less convincing, as it is not known if there are similar effects in depressed subjects. Chronic stress is a well-known method to induce animal models of depression [74]. It has been shown that BDNF expression was significantly reduced in the hippocampus of the chronic stressed-animals that exhibited depressive-like behaviors [75,76]. Hence, this animal model of depression could be an appropriate research model for studying the antidepressant effects of *H. erinaceus*. It would also be interesting to examine if *H. erinaceus* extract can restore BDNF levels and depressive behavior in chronic stressed-animals. The underlying mechanisms of these effects would be worth investigating.

**Table 1:** The behavioral and physiological effects of different *Hericium erinaceus* extracts in the studies of depression.

Types of Study	Authors	Material Studied	Method of Extraction	Dose and Dosage	Research Model	Behavioural Effects	Physiological Effects/Mechanism
**Pre-clinical**	Yao et al., 2015 [33]	Amycenone^®^, *H. erinaceus* fruiting body extract (0.5% hericenones and 6% amyloban)	Patented extraction	50, 100, or 200 * mg/kg amycenone (Amyloban^®^ 3399), administered 60 min prior to 0.5 mg/kg LPS injection; P.O.	Male C57BL/6N *mus musculus* (LPS-induced inflammation model of depression)	Anti-inflammatory and antidepressant-like effects	Attenuate a rise in the serum TNF-α level induced by LPS Increase the serum IL-10 level induced by LPS
	Ryu et al., 2017 [32]	*H. erinaceus*	Ethanolic extract	10, 60 * mg/kg daily for 4 weeks; P.O.	Male C57BL/6 *mus musculus*	Antidepressant-like and anxiolytic effects	Increase PCNA+, Ki67, BrdU+ cells. Hippocampal neurogenesis.
	Chiu et al., 2018 [31]	Erinacine A enriched *H. erinaceus* mycelium	Ethanolic extract	100, 200 *, and 400 * mg/kg daily for 4 weeks; P.O.	50 (10/group) male ICR *mus musculus* (14 days restraint stress induced model of depression)	Antidepressant-like effects	Induce BDNF/TrkB/PI3K/Akt/GSK-3β pathways. Inhibit NF-κB signalling Reduced IL-6 and TNF-α levels Increase 5-HT, DA, NE levels
**Clinical**	Nagano et al., 2010 [77]	*H. erinaceus* fruiting body	Water extract	500 * mg powdered fruiting body of *H. erinaceus* (Aso Biotech Inc) per cookie, 4 cookies daily for 4 weeks; P.O.	30 female participants	Alleviate symptoms of depression and anxiety	N.A.
	Inanaga, 2014 [78]	Amycenone^®^, *H. erinaceus* fruiting body extract (0.5% hericenones and 6% amyloban)	Patented extraction	1950 mg/tablet (Amyloban^®^ 3399) 6 tablets, divided into 2 or 3 doses /day for 6 months; P.O.	1 male patient	Improve neurocognitive impairment	N.A
	Okamura et al., 2015 [79]	Amycenone^®^, *H. erinaceus* fruiting body extract (0.5% hericenones and 6% amyloban)	Patented extraction	1950 mg/tablet (Amyloban^®^ 3399) 6 tablets, divided into 2 or 3 doses /day for 4 weeks; P.O.	8 female healthy participants	Alleviate symptoms of depression and anxiety Alleviate sleep disorders	Increase salivary levels of free-MHPG
	Vigna et al., 2019 [80]	*H. erinaceus* (80% mycelia and 20% fruiting body)	Water and ethanolic extract	1200 * mg per capsules (A.V.D. Reform s.r.l.), 3 capsules/day for 8 weeks; P.O.	62 females and 15 males overweight or obese participants	Alleviate symptoms of depression and anxiety Alleviate sleep disorders	Increase circulating pro-BDNF level without any significant change in BDNF circulating level

Indicator: * Dose of *H. erinaceus* with significant antidepressant-like effects.

A recent study by Chiu et al. (2018) investigated the effects of extracts of *H. erinaceus* enriched in Erinacine A (**5**) in an animal model of depression induced by repeated restraint stress [31]. They found that bioactive compounds extracted from the mycelium of *H. erinaceus* by ethanolic extraction were enriched with erinacine A, which is believed to induce neurogenesis. They showed that the extracts enriched with erinacine A reduced the immobility time in both the forced swim test and tail suspension test, indicating it had antidepressant-like effects. However, they did not detect an anxiolytic effect in the elevated plus-maze, which contradicted the previous findings by Ryu et al. in 2018 [32]. One possible reason for this discrepancy is that the two studies used different *H. erinaceus* extracts, as Chiu et al. (2018) used the ethanolic extract enriched with erinacine A extracted from the mycelium [31], whereas Ryu et al. (2018) used the ethanolic extract from the fruiting body [32]. Further research on the anxiolytic effects of *H. erinaceus* is required to confirm these findings. The use of extracts from *H. erinaceus* mycelium enriched with erinacine A may be advantageous, as erinacine A was reported to enhance nerve growth factor (NGF) activity to promote neurite outgrowth and its therapeutic effect was validated in the central nervous system of rats [81,82]. However, the use of these extracts from *H. erinaceus* may not represent the anti-depressant effects of *H. erinaceus* in its natural state. Furthermore, natural *H. erinaceus* contains many other erinacines, including erinacines A (**5**), B (**6**), C (**7**), D (**8**), E (**9**), F (**10**), and H (**12**), which have been found to also enhance NGF synthesis [81,83,84,85]. Higher doses of natural *H. erinaceus* may provide a similar effect to the erinacine A-enriched extract from *H. erinaceus* mycelium, and thus enrichment may not be necessary.

### 3.2. Clinical Studies

Prior to the in vivo studies that specifically looked at the antidepressant-like effects of *H. erinaceus*, Nagano et al. (2010) studied the clinical effects of *H. erinaceus* on menopause, depression, sleep quality, and indefinite complaints through a structured questionnaire survey of Kupperman Menopausal Index, Center for Epidemiologic Studies Depression Scale, Pittsburgh Sleep Quality Index (PSQI), and Indefinite Complaints Index in 30 females with an average age of 41.3 years over the period of 4 weeks [77]. Their findings revealed that consumption of cookies containing 0.5 g of fruitbodies powder alleviated the symptoms of depression, anxiety, frustration, and palpitation. However, the conclusions are less convincing as the study was gender-specific by design as it was related to menopause and also because a small study population was used.

In 2014, Inanaga et al. reported an improvement in neurocognitive function after treatment with Amyloban^®^ 3399 (tablets of standardized extract) in an 86-year-old male patient with recurrent depressive disorder [78]. However, mirtazapine, an antidepressant drug was also administered together with Amyloban^®^ 3399. Thus, the antidepressant effects could not be fully assessed in this study whether the alleviation in mood was a result of mirtazapine or the Amyloban^®^ 3399 or both. Additionally, a pilot study by Okamura et al. (2015) demonstrated that administration of Amyloban^®^ 3399 on female undergraduate students with sleep disorder for 4 weeks revealed an increase in the salivary level of free 3-methoxy-4-hydroxyphenylglycol, a biological index of anxiety disorders, which corresponds to an improvement in anxiety and sleep quality [79]. Sleep quality and general health status were assessed by the General Health Questionnaire (GHQ-28) and PSQI. In this pilot study, only eight female undergraduate students were recruited and who were scheduled to take a national examination in about a month, the result is unconvincing as the studied population is small and gender-specific. Furthermore, sleep disorder, anxiety, and mood disorder could be just temporary effects associated with their preparation for the national examination, which is different from the severity of clinically diagnosed anxiety and mood disorders.

Recently, a clinical study examined the effects of *H. erinaceus* on anxiety, depression, binge eating, and sleep disorders in 77 volunteers with a body mass index (BMI) ≥ 25 kg/m^2^ and an average age of 53.2 [80]. The study recruited overweight or obese participants positive for one or more administered tests, including Zung’s Depression Self-Assessment Scale, Zung’s Anxiety Self-Assessment Scale, Symptom Checklist-90, and the binge eating scale (BES). Participants in the *H. erinaceus* intervention group received three capsules containing 80% mycelium extract and 20% fruiting body extract daily for 8 weeks. They found that *H. erinaceus* significantly reduced depression and anxiety, as well as improvement on sleep disorders after 8 weeks of oral administration. The observation was linked to an increase in peripheral pro-BDNF and in the pro-BDNF/BDNF ratio. However, it was not clear whether these behavioral results might be partly due to a placebo effect from consuming a capsule. The experimental design of this study could have been improved by including placebo capsules in the control group and increasing the sample population. Although these studies showed that *H. erinaceus* has anti-depressant effects in female patients with symptoms of menopause and in obese patients, a clinical study on the antidepressant effects of *H. erinaceus* has yet to be conducted in the general depression population with and without gender bias.

## 4. Bioactive Compounds of *H. erinaceus* that Contribute to Antidepressant-Like Activities

Fruiting bodies and mycelia of *H. erinaceus* contain a variety of structurally diverse bioactive compounds that can induce the expression of various neurotrophic factors [81,83,84,86] and monoamines [31], and modulate inflammatory response [33]. At present, most of the identified bioactive compounds that contribute to antidepressant-like effects are mostly associated with NGF-inducing activity. The bioactive compounds of *H. erinaceus* that affect NGF release can be narrowed down to hericenones and erinacines. The small molecular sizes of hericenones and erinacines allow them to pass easily through the blood–brain barrier. These two major bioactive compounds have been investigated in most of the studies.

### 4.1. Hericenones

**Figure 3:**
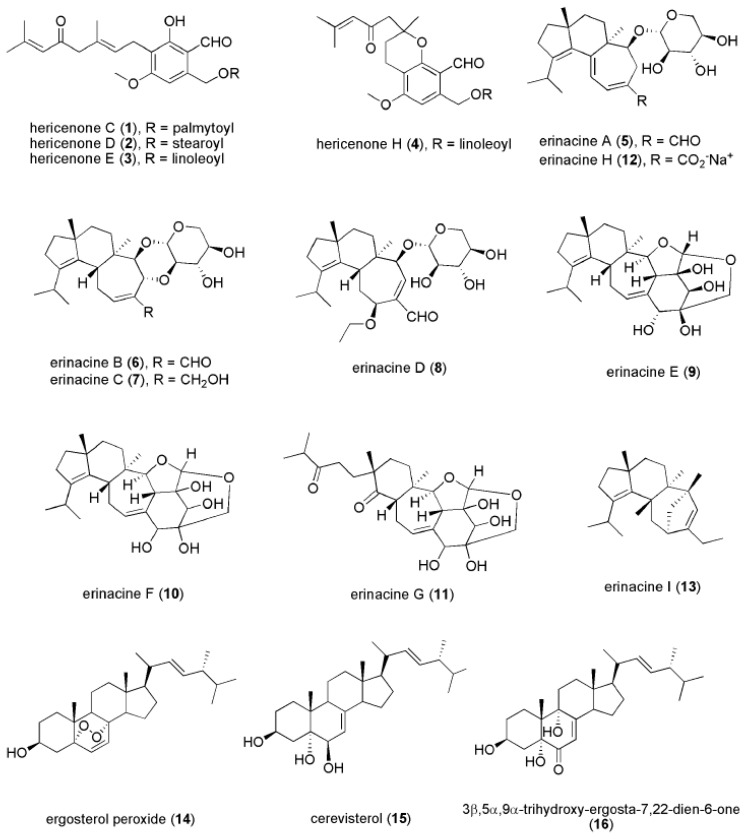
Chemical structures of hericenone C (**1**), D (**2**), E (**3**), H (**4**), erinacine A (**5**), H (**12**), B (**6**), C (**7**), D (**8**), E (**9**), F (**10**), G (**11**)**,** I (**13**), ergosterol peroxide (**14**), cerevisterol (**15**), and 3β,5α-trihydroxy-ergosta-7,22-dien-6-one (**16**).

Hericenones are aromatic compounds extracted from the fruiting body of *H. erinaceus.* There are 11 hericenones (hericenones A-K) that have been identified, of which four (hericenones C (**1**), D (**2**), E (**3**), and H (**4**) (Figure 3)) have been reported to promote NGF synthesis in mouse astrocytoma cells [86,87]. Mouse astroglial cells secreted 23.5 ± 1.0, 10.8 ± 0.8, 13.9 ± 2.1, and 45.1 ± 1.1 pg/mL NGF after treatment with 33 μg/mL hericenones C, D, E, and H, respectively. However, Mori et al. (2008) did not find that hericenones C, D, and E promoted NGF gene expressions at 10–100 mg/mL in 1321N1 human astrocytoma cells [88]. This raises the possibility that the NGF-promoting activity involves other bioactive compounds besides hericenones. Further in vivo studies on hericenones are needed to examine its effectiveness in stimulating NGF synthesis to resolve these inconsistent in vitro findings.

### 4.2. Erinacines

Erinacines have been mostly isolated from mycelium of *H. erinaceus*, however, erinacines A (**5**) and B (**6**) can also be found in the fruiting bodies [89]. Erinacines belong to a group of cyathin diterpenoids and have been shown to induce NGF synthesis. Currently, 15 erinacines have been identified, including erinacines A–K, P, Q, and S [81,82,84,85,90,91,92]. Among these erinacines, erinacines A-I (**5**–**13**) (Figure 3) were found to promote NGF synthesis, although other erinacines are still being investigated [81,82,84,85,90,93]. However, the underlying mechanism of how erinacines enhance NGF release remains unclear.

### 4.3. Novel Compounds

Many novel bioactive compounds of *H. erinaceus* are being actively discovered [19,94]. Other than hericenones and erinacines, Zhang et al. (2015) found several newly identified compounds isolated from the fruiting body of *H. erinaceus*, including ergosterol peroxide (**14**), cerevisterol (**15**), and 3β,5α,9α-trihydroxy-ergosta-7,22-dien-6-one (**16**) (Figure 3), which exhibited NGF-inducing activity and promoted neurite outgrowth in in vitro assays [23]. Additionally, amycenone isolated from the fruiting body of *H. erinaceus* exhibited anti-inflammatory activity that alleviated the inflammation-associated depression [33]. Of particular interest, the ethanolic extract of *H. erinaceus* mycelium enriched with erinacine A was found to modulate the expression level of serotonin, noradrenaline, and dopamine, as well as the BDNF signaling [31]. However, the bioactive compounds that contributed to the antidepressant-like effects remain to be identified.

## 5. Mechanism of Action

### 5.1. Stimulation of NGF and Proliferative Activities

Hippocampal neurogenesis is one of the major therapeutic targets for the treatment of depression based on the neurogenic hypothesis of depression. Bioactive compounds extracted from *H. erinaceus* including its mycelia and fruiting bodies were found to stimulate the expression of neurotrophic factors such as NGF [81,84,86,87]. Increased levels of NGF were found to be associated with neurogenesis and neuroplasticity [48], which may potentially lead to antidepressant-like effects. A study by Ryu et al. (2018) found that chronic administration of *H. erinaceus* ethanolic extract (60 mg/kg) significantly increased the number of PCNA-positive cells and Ki67-positive cells in the subgranular zone of the dentate gyrus, a region that consists of adult neural stem cells [32]. This result indicates that chronic high-dose *H. erinaceus* could promote the proliferation of hippocampal neural stem or progenitor cells. Furthermore, chronic administration of the *H. erinaceus* extract also increased the number of bromodeoxyuridine (BrdU)-immunoreactive cells in the granule cell layer of the dentate gyrus. Additionally, there was an increase in BrdU/NeuN double-labeled cell. These results indicate that chronic high-dose *H. erinaceus* could increase the number of mature hippocampal neurons differentiated from new neurons present prior to the first administration of *H. erinaceus.* Overall, these findings showed that chronic high-dose *H. erinaceus* treatment could promote hippocampal neurogenesis and increase the survival of new neurons in the dentate gyrus. The underlying mechanism of hippocampal neurogenesis induced by *H. erinaceus* administration was suggested to involve NGF synthesis. Nerve growth factor is necessary to regulate differentiation, proliferation, and maintenance of neuronal cells. Extracts of *H. erinaceus* were found to increase both NGF mRNA and protein expression in the hippocampus, indicating the bioactive compounds from *H. erinaceus* extract could pass through the blood–brain barrier leading to hippocampal neurogenesis [32]. It was previously reported that NGF levels were increased in the locus coeruleus and hippocampus of rats after receiving 8 mg/kg erinacine A [82]. This is in line with the in vitro finding that ethanolic extract from the fruiting body of *H. erinaceus* could promote neurite outgrowth in PC12 cells and stimulate NGF synthesis in 1321N1 human astrocytoma cells [88]. Mori et al. (2008) reported that *H. erinaceus* ethanolic extract with 100 µg/mL significantly increased the NGF mRNA and protein expression in 1321N1 human astrocytoma cells. They also found that 7 days of oral administration of 5% *H. erinaceus* dry powder increased *NGF* gene expression in the hippocampus of mice [88]. In 2008, Mori et al. treated 1321N1 cells with several kinase inhibitors followed by *H. erinaceus* ethanolic extract. *Hericium erinaceus* enhanced NGF activity, which was found to be inhibited by the c-Jun N-terminal kinase (JNK) inhibitor. The JNK serine-threonine protein kinase is involved in the phosphorylation of its downstream substrate c-Jun, a component of the activator protein 1 (AP-1) transcription factor [88]. *Hericium erinaceus* ethanolic extract promoted the phosphorylation of JNK and c-Jun, as well as the expression of c-Fos in vitro. These results further suggest that *H. erinaceus* ethanolic extract enhances NGF synthesis through the JNK pathway.

### 5.2. Monoaminergic Modulation

Modulation of monoamine neurotransmitters is another major therapeutic target for the treatment of depression. Chiu et al. (2018) showed that 14 days of restraint stress reduced the levels of monoamines neurotransmitters in the hippocampus of mice. Interestingly, the chronic administration of high-dose (400 mg/kg) *H. erinaceus* mycelium extract effectively restored the depleted expression levels of serotonin, norepinephrine, and dopamine in the hippocampus of restraint stressed-animals [31]. These results suggest that *H. erinaceus* has anti-depressant-like effects through serotonergic, noradrenergic, and dopaminergic modulations in restraint stressed animals. However, this finding raised the question that how does *H. erinaceus* modulate the concentration of the monoamine neurotransmitters. The detailed modulation pathway remains unknown and needed to be further investigated whether the bioactive compound of *H. erinaceus* acts as an MAO inhibitor which inhibits the enzymatic degradation of MAO thus preventing the reduction of monoamine neurotransmitters.

### 5.3. Anti-Inflammatory Pathway

To examine the anti-inflammatory effects of amycenone isolated from *H. erinaceus* fruiting body extract, the LPS-induced inflammation model of depression was pre-treated with amycenone 1 h before the intraperitoneal injection of LPS [33]. Blood was collected 90 min after the LPS injection for the measurement of serum TNF-α and IL-10 levels. Acute oral administration of 50, 100, and 200 mg/kg amycenone was found to attenuate the rise in serum TNF-α level induced by LPS, and 200 mg/kg amycenone significantly increased the rise in serum IL-10 level induced by LPS injection. Both TNF-α and IL-10 were previously reported to be associated with depression, in which TNF-α is a pro-inflammatory cytokine, while IL-10 is an anti-inflammatory cytokine [71,95,96]. The attenuation of the rise in TNF-α level and the enhanced-upregulation of IL-10 by acute treatment of amycenone suggested that the antidepressant-like effects of amycenone were through anti-inflammatory pathway. However, the detailed molecular mechanisms of action are still needed to be investigated.

Additionally, chronic oral administration of 200 and 400 mg/kg *H. erinaceus* mycelium effectively inhibited the increase in hippocampal expression levels of IL-6 and TNF-α induced by a paradigm of chronic restraint stress in a mouse model [31]. This result suggests that the anti-depressant effect of *H. erinaceus* involves the modulation of the inflammatory pathway. Related to an inflammatory response, erinacine A isolated from *H. erinaceus* was also reported to have neuroprotective effects against 1-methyl-4-phenyl-1,2,3,6-tetrahydropyridine (MPTP)-induced neurotoxicity through oxidative stress signaling and activation of the IRE1α/TRAF2, JNK1/2, and p38 MAPK pathways [29]. Although an anti-inflammatory response was demonstrated after *H. erinaceus* treatment, the detailed underlying molecular mechanisms remain unclear. Chiu et al. (2018) showed that chronic administration of *H. erinaceus* was able to restore the expression levels of nuclear factor-kappa B (NF-κB) and IκB that were reduced in the hippocampus of animals subjected to chronic restraint stress [31]. NF-κB is an important transcription factor in chronic inflammatory diseases, which is involved in the expression of various proinflammatory genes such as chemokines, cytokines, and adhesion molecules [97]. Based on these findings, the anti-depressant effects of *H. erinaceus* could be through an anti-inflammatory response via modulating the expression levels of IL-6, TNF-α, and NF-κB.

### 5.4. BDNF Pathway

Other than the NGF pathway, *H. erinaceus* mycelium was also reported to activate BDNF/TrkB/PI3K/Akt/GSK-3β pathways and inhibit NF-κB signaling in mice [31]. Chronic administration of *H. erinaceus* ethanolic extract was found to normalize the expression levels of BDNF, TrkB, and PI3K that were downregulated in animals with chronic restraint stress [31]. In addition, chronic administration of *H. erinaceus* inhibited the reduced expression levels of Akt-p and GSK-3β-p, but not Akt and GSK-3β, in the hippocampus of mice induced by repeated restraint stress. Similarly, erinacine C (**1**) isolated from *H. erinaceus* mycelium was reported to increase BDNF expression in 1321N1 cells [98]. The restoration effect of *H. erinaceus* on BDNF was suggested to be through monoaminergic modulation and normalization, as BDNF can be influenced by monoaminergic transmitters such as serotonin, norepinephrine, and dopamine [99]. A recent clinical study on patients with depression, anxiety, and sleep disorder examined the beneficial effects of 8 weeks of oral administration of capsules containing *H. erinaceus* extract (1200 mg/capsule, 3 capsules/day) [80]. They reported that pro-BDNF levels in the blood serum increased after 4 weeks of *H. erinaceus* administration, but there were no significant changes in serum BDNF levels. However, serum BDNF levels decreased after 8 weeks of *H. erinaceus* administration. The detailed underlying mechanism of the role of BDNF expression in the anti-depressant effects of *H. erinaceus* remains unclear.

**Figure 4:**
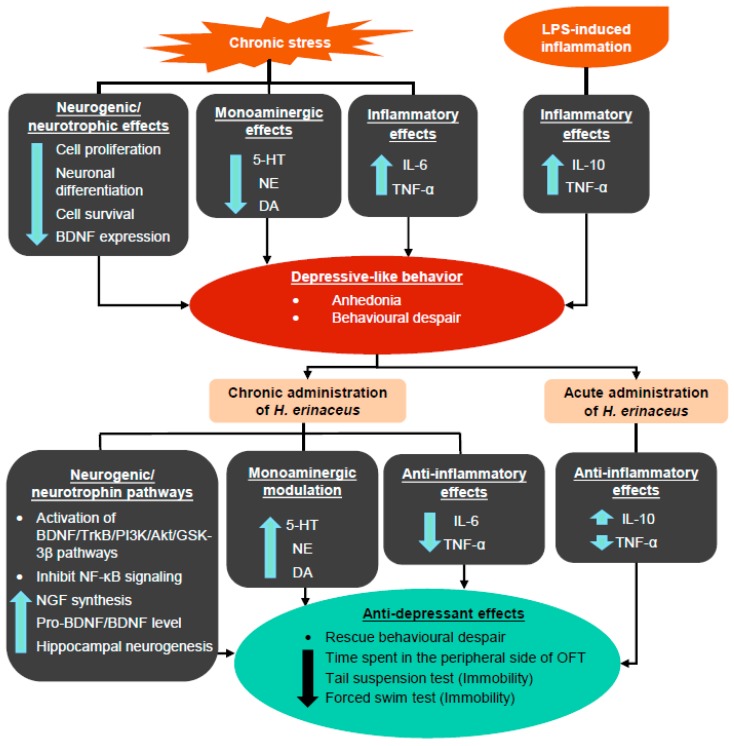
Summary of the generation of depressive-like behaviors induced by chronic stress and LPS-induced inflammation, as well as the mechanisms of antidepressant-like effects induced by *H. erinaceus.* The up arrow indicates an increase in the activity/expression level, while the down arrow indicates a decrease in the activity/expression level.

Overall, these results suggest that *H. erinaceus* ameliorates depressive-like behavior through the modulation of monoamine neurotransmitters and proinflammatory cytokines, as well as through the activation of BDNF pathways (Figure 4).

## 6. Future Perspectives of *H. erinaceus* Research in Depressive Disorder

*Hericium erinaceus* crude extract contains various hericenones, erinacines, and possibly other bioactive compounds that are still being discovered. Overall, the potent NGF-enhancing activities of *H. erinacines* are possibly mediated through the synergistic effects of several compounds in the crude extract. These compounds can greatly enhance adult hippocampal neurogenesis and contribute to the antidepressant-like effects. Chronic stress is known to induce an anhedonia effect that is highly associated with depression [100,101]. However, the anti-anhedonia activity of *H. erinaceus* has not been reported yet and needs to be investigated in future studies. Furthermore, it would be interesting to examine if hericenones, erinacines, or/and other compounds of *H. erinaceus* can be absorbed in the blood and pass through the blood–brain barrier. The variety of bioactive compounds with potent NGF-inducing activity also supports the use of crude extract rather than pure extracts. The majority of studies on *H. erinaceus* bioactive compounds have focused on their NGF activities. Future studies on the antidepressant activity of these bioactive compounds need to examine their effects on the expression of BDNF. Although BDNF was found to be restored in the hippocampus after chronic administration of *H. erinaceus*, it is still unclear whether the increase is also reflected in peripheral BDNF expressions [80]. The investigation of the effects on peripheral BDNF expression may provide important information for future clinical studies of depression. Moreover, as chronic administration of *H. erinaceus* was found to increase peripheral pro-BDNF but not BDNF [80], it would be interesting to examine the association of neurotrophic isoforms with depressive-like behaviors and whether *H. erinaceus* also affects their expression. In addition, as *H. erinaceus* was shown to stimulate monoaminergic modulation, it would be interesting to examine if the bioactive compounds of *H. erinaceus* act as agonists or inhibitors of monoamine neurotransmitter receptors. Although treatment with *H. erinaceus* was found to elicit an anti-inflammatory response, the detailed molecular mechanism is still unknown. These studies provide evidence that *H. erinaceus* possesses potential in alleviating depression, but the precise underlying mechanisms remain to be investigated. However, there is still a lack of strong and convincing evidence that *H. erinaceus* can effectively reduce anxiety and depression in vivo, which requires further investigation. Moreover, compounds in the extracts from the mycelium and fruiting body, as well as the method of extraction, requires further investigation to optimize the efficacy of *H. erinaceus* as a treatment for depressive disorders. In present investigation, majority of the studies does not include placebo or positive controls; and therefore, conventional antidepressants should be included as a positive control in future placebo-controlled research to compare their efficacy and eliminate potential placebo or non-specific effect. Furthermore, there is no concrete evidence of bioactive compounds unique to *H. erinaceus* that are responsible for its therapeutic effects, therefore future investigations involving selected medicinal-culinary mushrooms are highly warranted to rule out placebo and/or general effects.

## 7. Conclusions

The pre-clinical and clinical studies have demonstrated that *H. erinaceus* significantly ameliorates depressive disorder through monoaminergic modulation, neurogenic/neurotrophic, and anti-inflammatory pathways, indicating the potential role of *H. erinaceus* as complementary and alternative medicine for the treatment of depression. Nevertheless, the current research on antidepressant effects by *H. erinaceus* is relatively still at an early stage, and the specific mechanisms underlying the antidepressant-like activities require further investigation.
